# Supplementary figures and images for: The effects of arbuscular mycorrhizal fungi and root interaction on the competition between Trifolium repens and Lolium perenne
Source: PeerJ. 2017 Dec 20;5:e4183. doi: 10.7717/peerj.4183 (PMC5741977; doi:10.7717/peerj.4183)

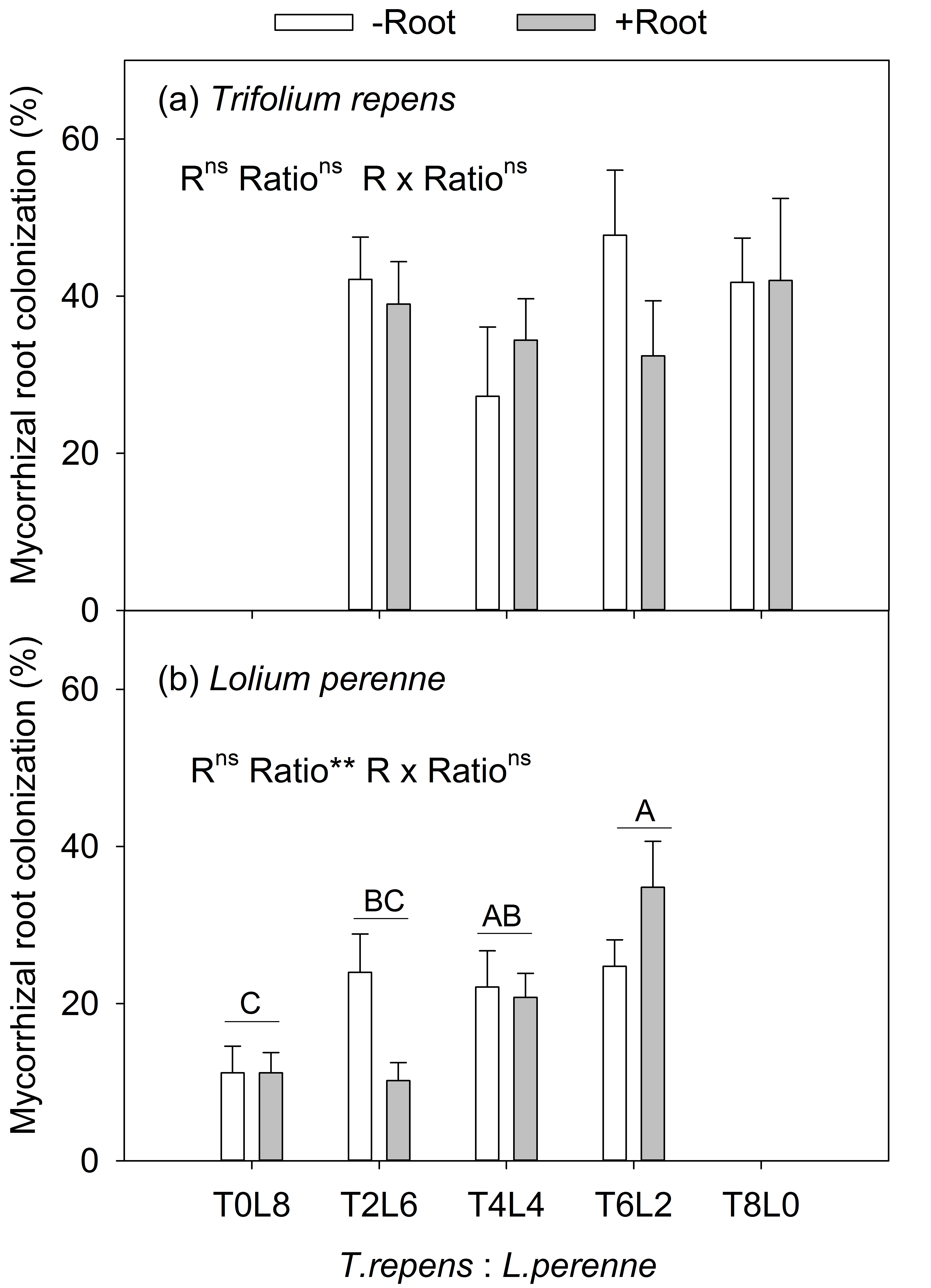

Supplement: Supplemental Information 1 — T8L0, T6L2, T4L4, T2L6, T0L8 mean planting ratio: 8:0, 6:2, 4:4, 2:6, 0:8, respectively. Bar groups with different letters indicate significant differences among planting ratios (n = 10 or 13). Data are means ± SE. **P < 0.01; ns P > 0.05. [file peerj-05-4183-s001.png]
