# Supplementary material for: The effects of arbuscular mycorrhizal fungi and root interaction on the competition between Trifolium repens and Lolium perenne
Source: PeerJ. 2017 Dec 20;5:e4183. doi: 10.7717/peerj.4183 (PMC5741977; doi:10.7717/peerj.4183)
Supplement: Supplemental Information 2 — Notes: Significant effects of treatments are indicated in bold. [file peerj-05-4183-s002.docx]

**Table S1** *F* ratios and *P* values resulting from GLM analysis of the effects of AMF inoculation (AMF) root interaction (R), planting ratio (Ratio) and their interactions on the shoot and root biomass of *T. repens* and *L. perenne*.

|  |  | Shoot biomass of  *T. repens* | |  | Shoot biomass of  *L. perenne* | | |
| --- | --- | --- | --- | --- | --- | --- | --- |
| Source of variation | df | *F* | *P* |  | | *F* | *P* |
| AMF | 1 | 0.08 | 0.7826 |  | | 0.21 | 0.6512 |
| R | 1 | **75.69** | **<.0001** |  | | **39.19** | **<.0001** |
| Ratio | 3 | **188.79** | **<.0001** |  | | **151.56** | **<.0001** |
| AMF*R | 1 | 0 | 0.9695 |  | | 0.01 | 0.9249 |
| AMF*Ratio | 3 | 1.05 | 0.3743 |  | | 0.71 | 0.5512 |
| R*Ratio | 3 | **6.38** | **0.0006** |  | | **13.32** | **<.0001** |
| AMF*R*Ratio | 3 | 1.5 | 0.2203 |  | | 0.05 | 0.9867 |
| Error | 88 |  |  |  | |  |  |
|  |  | Root biomass of  *T. repens* | |  | | Root biomass of  *T. repens* | |
| Source of variation | df | *F* | *P* |  | | *F* | *P* |
| AMF | 1 | 2.92 | 0.0909 |  | | 0.14 | 0.7115 |
| R | 1 | **4.52** | **0.0363** |  | | **125.71** | **<.0001** |
| Ratio | 3 | **191.97** | **<.0001** |  | | **47.76** | **<.0001** |
| AMF*R | 1 | 0.8 | 0.3721 |  | | 0 | 0.979 |
| AMF*Ratio | 3 | 0.4 | 0.7554 |  | | 0.3 | 0.8277 |
| R*Ratio | 3 | **9.47** | **<.0001** |  | | **3.97** | **0.0108** |
| AMF*R*Ratio | 3 | 0.42 | 0.7385 |  | | 1.12 | 0.3451 |
| Error | 88 |  |  |  | |  |  |

*Notes*: Significant effects of treatments are indicated in bold.
