# Supplementary material for: The effects of arbuscular mycorrhizal fungi and root interaction on the competition between Trifolium repens and Lolium perenne
Source: PeerJ. 2017 Dec 20;5:e4183. doi: 10.7717/peerj.4183 (PMC5741977; doi:10.7717/peerj.4183)
Supplement: Supplemental Information 3 — Significant effects of treatments are indicated in bold. *p < 0.05; ***p < 0.0001. [file peerj-05-4183-s003.docx]

**Table S2** *F* ratios resulting from GLM analysis of the effects of AMF inoculation (AMF) root interaction (R), planting ratio (Ratio) and their interactions on the relative yield per individual (RY_ind_) of *T. repens* and *L. perenne* and the relative yield total (RYT) in mixtures.

| Source of variation | df | RY_ind_ of  *T. repens* | RY_ind_ of  *L. perenne* | df | RYT |
| --- | --- | --- | --- | --- | --- |
| AMF | 1 | 0.18 | 0.01 | 1 | 0.09 |
| R | 1 | **27.78***** | **43.86***** | 1 | 0.35 |
| Ratio | 3 | **25.47***** | **367.97***** | 4 | **11.13***** |
| AMF*R | 1 | 0.5 | 0.02 | 1 | 0.06 |
| AMF*Ratio | 3 | 1.43 | 0.5 | 4 | 0.22 |
| R*Ratio | 3 | **3.15*** | **26.08***** | 4 | 0.08 |
| AMF*R*Ratio | 3 | 1.53 | 0.03 | 4 | 0.7 |
| Error | 88 |  |  | 104 |  |

*Notes*: Significant effects of treatments are indicated in bold. **p* < 0.05; ****p* < 0.0001.
