# Supplementary material for: The effects of arbuscular mycorrhizal fungi and root interaction on the competition between Trifolium repens and Lolium perenne
Source: PeerJ. 2017 Dec 20;5:e4183. doi: 10.7717/peerj.4183 (PMC5741977; doi:10.7717/peerj.4183)
Supplement: Supplemental Information 4 — Significant effects of treatments are indicated in bold. **p < 0.01; *** p < 0.0001. [file peerj-05-4183-s004.docx]

**Table S3** *F* ratios resulting from GLM analysis of the effects of AMF inoculation (AMF) root interaction (R), planting ratio (Ratio) and their interactions on the shoot N and P content of *T. repens* and *L. perenne*.

|  |  | N% | | |  | P% | | |
| --- | --- | --- | --- | --- | --- | --- | --- | --- |
| Source of variation | df | *T. repens* | *L. perenne* | df | | | *T. repens* | *L. perenne* |
| AMF | 1 | 1.99 | 0.15 | 1 | | | 0.73 | 1.26 |
| R | 1 | 0.18 | 0.14 | 1 | | | 0.86 | 0 |
| Ratio | 3 | 0.48 | **14.21***** | 3 | | | 2.07 | 0.4 |
| AMF*R | 1 | 0.39 | 3.41 | 1 | | | 1.92 | 0.33 |
| AMF*Ratio | 3 | **6.10**** | 2.56 | 3 | | | 0.36 | 0.21 |
| R*Ratio | 3 | 1.86 | 0.24 | 3 | | | 0.07 | 0.56 |
| AMF*R*Ratio | 3 | 1.52 | 1.15 | 3 | | | 3.07 | 1.46 |
| Error df |  | 88 | 83 |  | | | 59 | 56 |

*Notes*: Significant effects of treatments are indicated in bold. ***p* < 0.01; *** *p* < 0.0001.
